# Evaluation of the melatonin and oxidative stress markers level in serum of fertile and infertile women

**Published:** 2015-07

**Authors:** Sara Soleimani Rad, Shamsi Abbasalizadeh, Amir Ghorbani Haghjo, Mehzad Sadagheyani, Azadeh Montaseri, Jafar Soleimani Rad

**Affiliations:** 1 *Department of Obstetrics and Gynecology, Alzahra Hospital, Faculty of Medicine, Tabriz University of Medical Sciences, Tabriz, Iran.*; 2 *Women's Reproductive Health Research Center, Tabriz University of Medical Sciences, Tabriz-Iran.*; 3 *Department of Medical Biochemistry, Faculty of Medicine, Tabriz University of Medical Sciences, Tabriz, Iran.*; 4 *Department of Anatomical Sciences, Faculty of Medicine, Tabriz University of Medical Sciences, Tabriz, Iran.*

**Keywords:** *Oxidative stress*, *Infertility*, *Melatonin*, *Malondialdehyde*, *total antioxidant capacity*

## Abstract

**Background:**

Infertility is defined as the inability to achieve the pregnancy within a year of unprotected intercourse. Infertility is a complex issue and different factors such as stress oxidative can be involved in this problem. So, any attempt to neutralize oxidative stress would be helpful in the treatment of infertility. Melatonin is a known scavenger of free radicals.

**Objective:**

The aim of our study was to evaluate the level of melatonin and its correlation with oxidative biomarkers in fertile and infertile women.

**Materials and Methods:**

The participants including fertile and infertile women were divided into two groups of 30 people. Blood sampling was performed and sera were collected. The level of Malondialdehyde (MDA), total antioxidant capacity (TAC) and melatonin were detected. Data were analyzed using T-test and their correlation was assessed using Spearman test.

**Results:**

Serum melatonin from fertile women was higher than infertile women but the difference was not significant (p= 0.46). MDA level in fertile women was significantly lower than infertile women (p<0.001) and the level of TAC in fertile women was significantly higher than infertile women (p<0.001). Spearman test revealed a significant and direct correlation between melatonin and TAC in fertile and infertile women and a significant but reverse correlation between melatonin and MDA in infertile and fertile women.

**Conclusion:**

Differences in the level of oxidative stress biomarkers in fertile and infertile individuals have been reported. This study revealed a significant correlation between melatonin and oxidative stress biomarkers, concluding that melatonin level could be involved in infertility.

## Introduction

Infertility is defined as the inability to achieve the pregnancy within a year of unprotected intercourse. Fifteen percent of young couples in different societies suffer from infertility (1). Infertility is a multifaceted issue with increasing prevalence in both developed and developing countries. Due to its importance, many researchers try to assist this matter and promote the health status of families and society (2). Increasing of our knowledge about the factors affecting fertilization, would be helpful in solving this problem (2).

Oxidative stress (OS) is a recognized factor with ability to affect human fertility. Free radicals are continuously produced as byproducts under aerobic cellular metabolism and play a key role in physiological and pathological processes. Antioxidant defense system assists the organism to respond and fight against the excess amount of free radicals (3). Overproduction of free radicals such as reactive oxygen species (ROS) or failure in antioxidant defense system results in OS (4), which is involved in several pathological conditions. The impact of OS in decreasing the egg quality, fertilization and pregnancy rates in mouse and human has been previously reported. On the other hand, it is reported that antioxidant depletion leads to infertility and use of antioxidants could be helpful in the treatment of infertility (5).

As the principal pineal hormone, melatonin plays a key role in regulating the function of neuroendocrine system. Melatonin is produced in a circadian rhythm with a maximal secretion at night (3). Melatonin plays an important role in the regulation of the hypothalamic-pituitary-gonadal axis in human (4). This hormone influences human ovarian and reproductive functions and high affinity binding sites for melatonin agonist has been detected on human granulosa cells membrane (6). As an effective antioxidant, melatonin detoxifies the free radicals and its presence is crucial for optimal cell and organ functions, including reproductive system (7). However, the melatonin level in infertile women and its correlation with OS biomarkers have not been established. Regarding to the role of melatonin level in infertility and lack of precise study in this field, the goal of the present study was to determine the correlation between melatonin and OS biomarkers in fertile and infertile women.

## Materials and methods

This cross-sectional analytical study was carried out in Alzahra obstetric and gynecology hospital, Tabriz- Iran, from Jan 2012 Jan 2013. The proposal was approved in ethic committee of the Tabriz university of Medical Sciences and registered as 5.4.8622-90.11.6. Accordingly, the consent was obtained from volunteers and the aim of the study was explained for every participant.

The fertile women have been considered as control group and the infertile women have been considered as case group. Sample size was chosen according to prevalence of infertility by consultation with a statistician. Thus, 30 cases for each group were determined.

The control group was selected from patients that referred to Alzahra hospital for any gynecological reasons other than infertility and had child during last one year. The infertile women have been chosen from the patients who were referred to Alzahra hospital for treatment of infertility. These women with unexplained infertility, without having tubal factor andor PCO, did not achieve pregnancy after one year with unprotected intercourse and their husbands had not any indication of infertility and had normal spermogram according to WHO criteria (1). Inclusion criteria in addition to being fertile or infertile were included: fertile age (15-45) and having of normal, up to 25 Body Mass Index.

**Blood sampling**

For hematological determinations, 5ml blood was obtained from each participant, transferred to sterile tube, centrifuged and their serum was separated for measurement of Malondialdehyde (MDA) and total antioxidant capacity (TAC), as OS biomarkers, and melatonin. For having a similar condition, blood sampling in all patients was restricted to about 10 AM. The sera were kept at – 80 degree Centigrade refrigerator until the time of measurement.

The MDA measurement was carried out according to Bilic et al. (8). The measurement was based on thiobarbituric acid (TBA) reaction and extraction with normal bothanol, spectrophotometric detection of absorbance at 532 wave length and comparing with standard curve. TBA was purchased from Merck.

TAC measurement in serum was carried out according to Miller et al. (9) using Randox kit. Melatonin measurement was carried out using human melatonin (MT) Elisa Kit, purchased from Glory company.

**Statistical analysis**

The data obtained for case and control groups were presented as Mean±SD and analyzed. For evaluation of association between MDA and TAC with melatonin, non parametric correlation Spearman test, SPSS.13.0 (Statistical Package for the Social Sciences, version 13.0, SPSS Inc, Chicago, Illinois, USA) was used. P<0.05 was considered as significant.

## Results

The average age of women in control group was 35.95±6.05 and in infertile women was 28.91±7.5. The average of BMI in control group was 26.35±2.97and in infertile women was 25.11±2.8. Hematologic parameters in both control and experiment groups were analyzed and the results are presented separately for each parameter.

**Serum level of melatonin**

The mean concentration of melatonin in the serum from fertile women was 519.33±52.41ng/L and in infertile women was 470.46±39.99ng/L. While the data showed that the melatonin level in infertile women was lower than that in fertile women however, the difference was not significant (p=0.46).

**Serum level of TAC**

The mean concentration of TAC in the serum from fertile women was 1.62±0.14nmol/L and in infertile men was 1.27±0.17nmol/L. Statistical analysis revealed a significant difference between two groups (p<0.001).

**Serum level of MDA**

The mean concentration of MDA in the serum from fertile women was 1.91±0.39 nmol/ml and in infertile women was 2.7±0.47nmol/ml. Statistical analysis revealed that the MDA level in fertile women was significantly (P<0.001) lower than those in infertile women.

**Correlation of melatonin, TAC and MDA**

**Figure 1:**
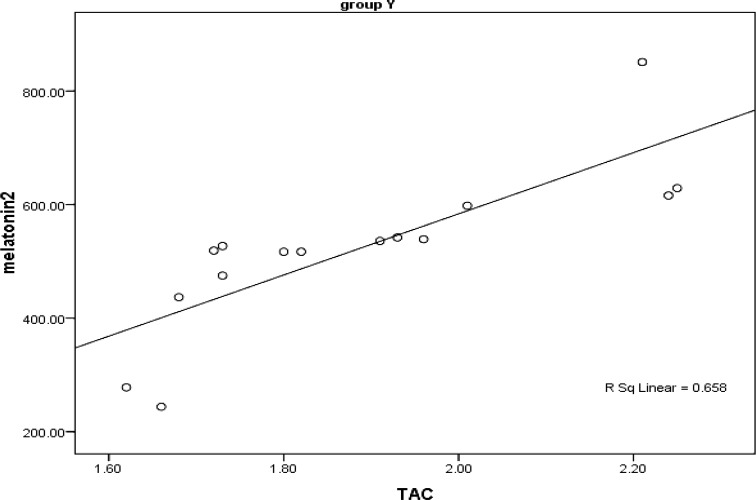
Scatter plat showing correlation of melatonin and TAC in fertile women

**Figure 2:**
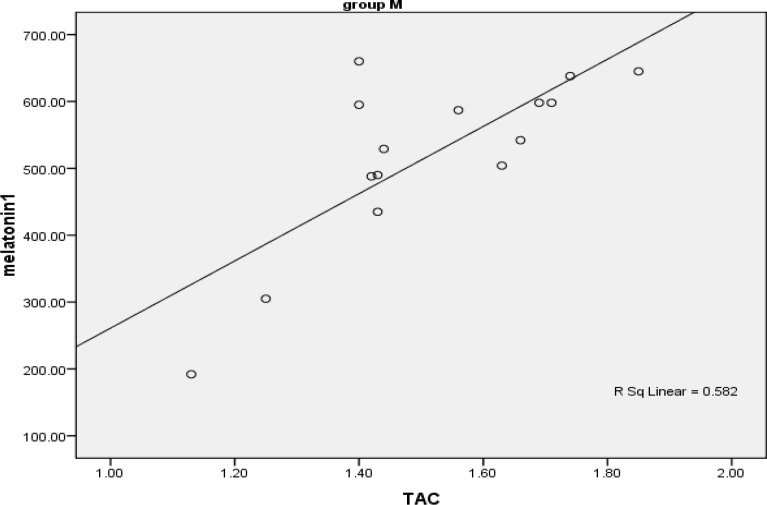
Scatter plat showing correlation of melatonin and TAC in infertile women

**Figure 3:**
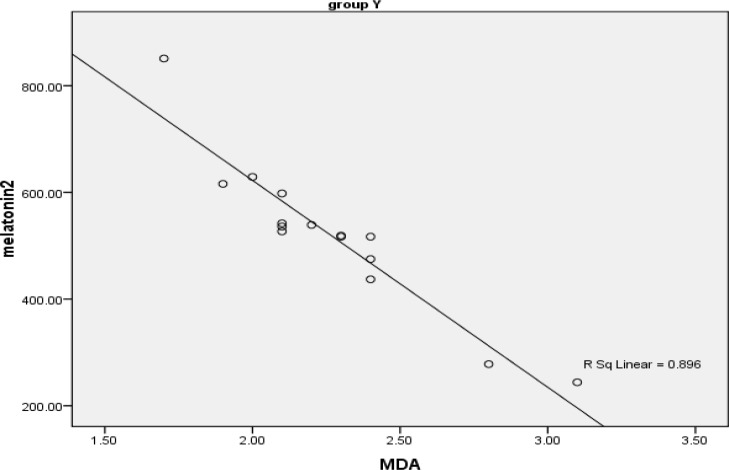
Scatter plat showing correlation of melatonin and MDA in fertile women

**Figure 4:**
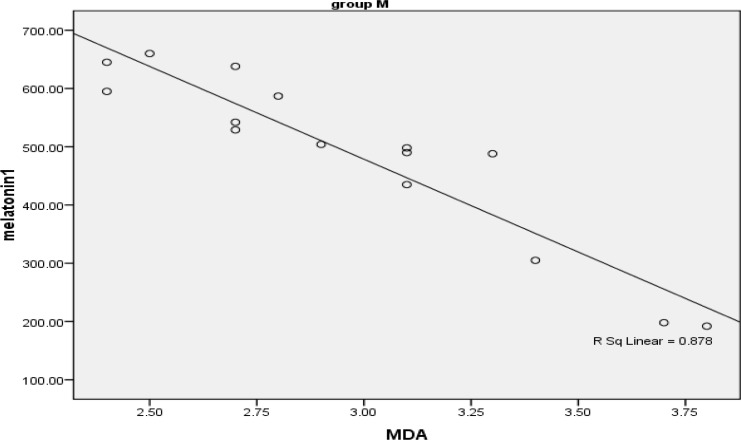
Scatter plat showing correlation of melatonin and MDA in infertile women

Figures 1-4 reveal correlation of melatonin with TAC and MDA. Spearman’s correlation coefficient ratio between TAC and melatonin in fertile women is presented in figure 1. As the figure shows there is a strong (rs=0.95) and significant (p<0.001) direct correlation between TAC and melatonin.

Figure 2 reveals the examination of the same parameters in infertile women. As the figure shows there is a strong (rs=0.95) and significant (P<0.001) direct correlation between TAC and melatonin in infertile women.

Figure 3 presents the Spearman’s correlation coefficient ratio between MDA and melatonin in fertile women. It can be found that there is a strong (rs= -0.89) and significant (p<0.001) negative correlation between MDA and melatonin in this group. The examination of the same parameters in infertile women is shown in figure 4. As the figure shows there is a moderate (rs= -0.57) and significant (p<0.01) negative correlation between MDA and melatonin in infertile women.

## Discussion

Understanding the factors that are involved in infertility would greatly be helpful in the treatment of infertility problem. The present study was carried out to investigate the possible role of melatonin as a free radical scavenger in infertility.

We found in the present study that: 1.The melatonin level in infertile women is lower than the fertile women, however this difference was not significant, 2. The TAC level in fertile women was significantly higher in comparison with infertile women, 3. The MDA level in fertile women was significantly lower than those in infertile women, 4. The correlation between TAC and melatonin in both fertile and infertile women were strong, direct and significant, 5. The correlation between MDA and melatonin in fertile and infertile women were strong, significant and reverse.

Physiology of the female reproductive system is affected by cellular reactive oxygen species (ROS) and antioxidants, as a defense system of the organism. It has been previously shown that the physiologic concentration of ROS plays a key role in folliculogenesis, oocyte maturation and uterine function (10) but its higher concentrations or organism's failure in combating ROS production (oxidative stress status) can initiate or develop the pathological conditions and affect reproductive processes (4).

The most important pineal gland hormone, melatonin, has direct and indirect antioxidant activity (11) and it can be detected in many body compartments. The function of ovary is controlled directly by melatonin. Previous studies revealed the presence of melatonin receptors on preovulatory follicular granulosa cells. Functions of granulosa cells such as steroidogenesis are altered in the presence of melatonin in hen, hamster and human (12).

Our study showed that the serum level of melatonin in infertile women was notably lower than the fertile woman, but this difference was not significant. The low level of melatonin in infertile women, in the present study, could still be defended in that; as the mean values of melatonin level show the level of melatonin ranges widely among the individuals. This is due to that melatonin is produced according to the circadian rhythm (3). On the other hand, the level of melatonin in human blood is affected greatly by varying numerous factors such as amount of light, stress, body position, physical activity, time of the day and so on (13). Although it is tried that demographic characteristic of participants such as BMI and age to be in a limited range and the time of blood collection was restricted to a limited time, but the control of other factors was almost impossible. In accordance with our findings, it has been demonstrated that melatonin regulates ovarian function through the regulation of gonadotropin release which is mediated via its specific receptors (14). It is also reported that, in human ovary, melatonin concentration in follicular fluid from preovulatory follicles is higher than in serum (15). Additionally, there is growing evidence that melatonin as an antioxidant, has a direct effect on oocyte maturation and embryo development (16). In vivo studies are consistent with in vitro findings in showing that melatonin administration in infertile patients reduced intrafollicular oxidative damage, increased fertilization and pregnancy rates during In vitro fertilization protocol (17). It is also believed that melatonin in the placenta plays an important role in regulating apoptotic processes and maintaining of optimal turnover of embryonic placental cells (18).

Our study also revealed that the serum level of TAC in infertile women was significantly (p<0.001) lower in comparison with fertile women and concentration of MDA in infertile women was significantly (p<0.001) higher in comparison with fertile women. The significant correlation between melatonin and OS factors indicates that low melatonin level in infertile women possibly is involved in production of OS.

In support of our findings, it has been previously reported that the excess amount of ROS, a main molecule involved in OS condition, impairs the human reproduction. It has been reported that the peripheral markers of OS significantly decreased in women with infertility due to different pathological conditions such as endometriosis (19).

Based on the evidence, ROS and antioxidant system are important in ovulation, fertilization, steroidogenesis and endometrial receptivity (15). Tamura et al. (2009) reported that the concentration of melatonin, as a free radical scavenger, is higher in the fluid of large follicles in comparison with its concentration in small size follicles (12). The role of melatonin in promoting fertilization rarte and embryonic development an maturation has been also revealed (16, 18). Furthermore, interaction of melatonin and its derivatives with free radicals and combatting OS is well known (20). These findings are in favor of our results in that in infertile women, the level of melatonin and TAC was lower in comparison with fertile women. As it has been previously described, human fertility is directly and indirectly controlled by OS.

Based on these documented data, we can conclude that melatonin as a scavenger of free radicals such as ROS, plays an important role in regulation of reproduction in female. It can also be suggested that the use of melatonin in infertility treatment could be taken into consideration.

## Note

***This article extracted from M.D. thesis. (Sara Soleimani Rad)***
